# A comprehensive nomogram combining CT-based radiomics with clinical features for differentiation of benign and malignant lung subcentimeter solid nodules

**DOI:** 10.3389/fonc.2023.1066360

**Published:** 2023-03-07

**Authors:** Chengyu Chen, Qun Geng, Gesheng Song, Qian Zhang, Youruo Wang, Dongfeng Sun, Qingshi Zeng, Zhengjun Dai, Gongchao Wang

**Affiliations:** ^1^ Department of Thoracic Surgery, Shandong Provincial Hospital, Cheeloo College of Medicine, Shandong University, Jinan, China; ^2^ Department of Thoracic Surgery, The First Affiliated Hospital of Shandong First Medical University, Jinan, China; ^3^ Department of Ultrasound, Shandong Provincial Hospital Affiliated to Shandong First Medical University, Jinan, China; ^4^ Department of Radiology, The First Affiliated Hospital of Shandong First Medical Unversity, Jinan, China; ^5^ Department of General Surgery, The First Affiliated Hospital of Shandong First Medical University, Jinan, China; ^6^ Elite Class of 2017, Shandong First Medical University, Jinan, China; ^7^ Scientific Research Department, Huiying Medical Technology Co., Ltd, Beijing, China

**Keywords:** radiomics, nomogram, CT, lung cancer, subcentimeter solid nodules

## Abstract

**Objective:**

To establish a nomogram based on non-enhanced computed tomography(CT) imaging radiomics and clinical features for use in predicting the malignancy of sub-centimeter solid nodules (SCSNs).

**Materials and methods:**

Retrospective analysis was performed of records for 198 patients with SCSNs that were surgically resected and examined pathologically at two medical institutions between January 2020 and June 2021. Patients from Center 1 were included in the training cohort (n = 147), and patients from Center 2 were included in the external validation cohort (n = 52). Radiomic features were extracted from chest CT images. The least absolute shrinkage and selection operator (LASSO) regression model was used for radiomic feature extraction and computation of radiomic scores. Clinical features, subjective CT findings, and radiomic scores were used to build multiple predictive models. Model performance was examined by evaluating the area under the receiver operating characteristic curve (AUC). The best model was selected for efficacy evaluation in a validation cohort, and column line plots were created.

**Results:**

Pulmonary malignant nodules were significantly associated with vascular alterations in both the training (p < 0.001) and external validation (p < 0.001) cohorts. Eleven radiomic features were selected after a dimensionality reduction to calculate the radiomic scores. Based on these findings, three prediction models were constructed: subjective model (Model 1), radiomic score model (Model 2), and comprehensive model (Model 3), with AUCs of 0.672, 0.888, and 0.930, respectively. The optimal model with an AUC of 0.905 was applied to the validation cohort, and decision curve analysis indicated that the comprehensive model column line plot was clinically useful.

**Conclusion:**

Predictive models constructed based on CT-based radiomics with clinical features can help clinicians diagnose pulmonary nodules and guide clinical decision making.

## Introduction

With increasing use of chest computed tomography(CT) in health check-ups and preoperative examinations, many pulmonary nodules that are difficult to identify on a chest X-ray are being detected (1). Common pulmonary nodules are usually classified into three categories: pure ground glass nodules (pGGNs), mixed ground glass nodules (mGGNs), and solid nodules (SNs) (2). Most solid nodules <1cm in diameter,also known as subcentimeter solid nodules (SCSNs), are benign and commonly encountered as intrapulmonary lymph, fibrous foci and tuberculosis nodules (3).But SCSNs do not guarantee their benignancy, some solid nodules are malignant, typically in lung adenocarcinoma (4, 5). Therefore, the diagnosis of pulmonary nodules is still important, since it is related to the need for close follow-up observation or surgical treatment. Early diagnosis can improve patient prognosis and reduce radiation exposure due to close follow-up (6). We found that there are more studies on pGGNs and mGGNs (7, 8), but less studies on SNs, especially SCSNs, so we focused on SCSN in an attempt to make a small contribution to the early diagnosis of lung cancer.

Nodule shape, margins, burr sign, and pleural pull sign can be used to determine the nature of the nodule. However, these signs are subjective and often not obvious when the nodules are small (2). Positron Emission Tomography-CT (PET-CT) is also commonly used for the diagnosis of tumors. It can aid in evaluating the metabolic activity of the mass to infer its nature, although its specificity for smaller lung nodules is low (9). Percutaneous biopsy is also used for the diagnosis of the nature of lung masses, but it has disadvantages, such as its invasive nature, which may lead to needle tract metastasis and puncture failure. It usually follows thoracoscopic resection biopsy as a last diagnostic resort (10, 11). Therefore, a more simple and accessible non-invasive and efficient diagnostic tool is needed.

Many researchers have realized that the morphological features on CT images do not provide a complete picture for diagnosis, and a large amount of high-dimensional and valuable data that cannot be seen are hidden in the images (12, 13). In 2012, Lambin et al. proposed using radiomics, which emphasizes extracting a large amount of information from images (CT, Magnetic Resonance Imaging, and PET-CT) in a high-throughput manner to achieve tumor image segmentation, feature extraction, and model building by virtue of deeper mining, analysis, and prediction of massive image data information to assist physicians in disease diagnosis, prognosis assessment, and treatment response prediction (14). Generally, the radiomics workflow consists of the following main steps: imaging data collection, imaging preprocessing, identification and segmentation of the region or volume of interest, feature extraction, feature selection, model establishment, and model validation (15, 16). Some studies have shown that chest CT radiomics can be used to predict benign and malignant lung nodules, aggressiveness, pathological typing, genetic phenotype, and treatment response and to determine prognosis (17–21). However, CT radiomics has not been applied to assess the malignancy of pulmonary SCSNs.

Therefore, the present study used non-enhanced chest CT imaging radiomics combined with clinical data to construct a predictive model for malignancy prediction of pulmonary SCSNs to guide clinical decision making.

## Materials and methods

This study was approved by the Ethics Committee of The First Affiliated Hospital of Shandong First Medical University (center 1) and the Shandong Provincial Hospital Affiliated to Shandong First Medical University (center 2). Patients’ informed consent was waived due to the retrospective nature of the study. A total of 147 patients hospitalized at center 1 between January 2020 and June 2021 were included in the modeling group. Moreover, 52 patients hospitalized at center 2 between January 2021 and June 2021 were included in the validation group. Inclusion criteria were as follows: a. SCSNs without obvious calcified components; b. thin-layer scans with layer thickness of ≤ 1.5 mm, c. CT images within two weeks before surgery, and d. patients underwent a complete surgical resection. Exclusion criteria were as follows: a. chemotherapy, radiotherapy, or previous history of malignancy prior to CT examination, b. poor image quality, and c. incomplete clinical or imaging data. Relevant patient information is shown in **Table 1**.

**Table 1:** Clinical characteristics and subjective CT findings of lung nodules in training and validation cohorts.

	Training Cohort					Validation Cohort				
Clinical parameters	Data	Benign	Malignant	χ^2^or t-value	P value	Data	Benign	Malignant	χ^2^or t-value	P value
Sex		82	65	0.696	0.494		31	21	0.027	0.870
Men	60	31	29			23	14	9		
Women	87	51	36			29	17	12		
Age	56.56 ± 10.95	55.38 ± 11.89	57.48 ± 10.12	3.385	<0.001	54.63 ± 12.61	52.19 ± 12.56	58.23 ± 11.55	-1.758	0.085
Smoke		82	65	4.712	0.030					0.687
Yes	27	10	17			7	5	2		
No	120	72	48			45	26	19		
Family history of lung cancer		82	65	0.536	0.464					0.639
Yes	13	6	7			4	3	1		
No	134	76	58			48	28	20		
DM		82	65	0.0355	0.853					0.558
Yes	12	7	5			3	1	2		
No	135	75	60			49	30	19		
NSE				0.158	0.691					0.683
Normal		58	44			47	28	19		
Abnormal		24	21			5	3	2		
CEA					0.184					0.645
Normal		79	59			49	29	20		
Abnormal		3	6			3	2	1		
SCC					0.023					0.291
Normal		80	57			48	30	18		
Abnormal		2	8			4	1	3		
Size	0.74 ± 0.21	0.74 ± 0.20	0.75 ± 0.21	0.905	0.343	0.89 ± 0.36	0.86 ± 0.38	0.93 ± 0.30	-0.675	0.503
Location				3.3841	0.496					0.689
RUL		31	20				6	7		
RML		3	5				4	1		
RLL		12	15				7	5		
LUL		20	15				5	2		
LLL		16	10				9	6		
Pleural indentation				25.3830	<0.001				4.461	0.035
Yes		16	39			23	10	13		
No		66	26			29	21	8		
Spiculation				5.006	0.025				7.188	0.007
Yes		7	14			23	9	14		
No		75	51			29	22	7		
Lobulation				4.378	0.036				2.632	0.105
Yes		2	7			18	8	10		
No		80	58			34	23	11		
Irregular air bronchogram				10.803	0.001					0.558
Yes		2	12			3	1	2		
No		80	53			49	30	19		
Cavitation sign				1.048	0.306					0.683
Yes		6	8			4	2	2		
No		76	57			48	29	19		
Vascular change				23.665	<0.001				20.974	<0.001
Yes		50	62			27	8	19		
No		32	3			25	23	2		

Differences were assessed using Student’s t-test and the chi-square test as appropriate; p < 0.05.

DM, diabetes mellitus; NSE, neuron-specific enolase; CEA, carcino-embryonic antigen; SCC, squamous cell carcinoma antigen; RUL, right upper lobe; RML, right middle lobe; RLL, right lower lobe; LUL, left upper lobe; LLL, left lower lobe.

### CT examination

CT examinations were performed using non-enhanced CT scanning on a high-definition CT (HDCT) system (GE Discovery CT750 HD; GE Healthcare, Milwaukee, WI, USA). The parameters were: tube current of 275 mA, tube voltage of 120 kV, helical scanning, 1.375 helical pitch, 0.7 s tube rotation time, pixel matrix of 512 × 512, adopting 40 mm collimation, 5 mm slice thickness, and 5 mm slice interval. Patients held their breath for scanning in the supine position. The scanning area covered the whole chest. Axial images were reconstructed with a slice thickness of 1.25 mm and an interval of 1.0 mm with a lung algorithm.

### Image feature evaluation

**Figure 1:**
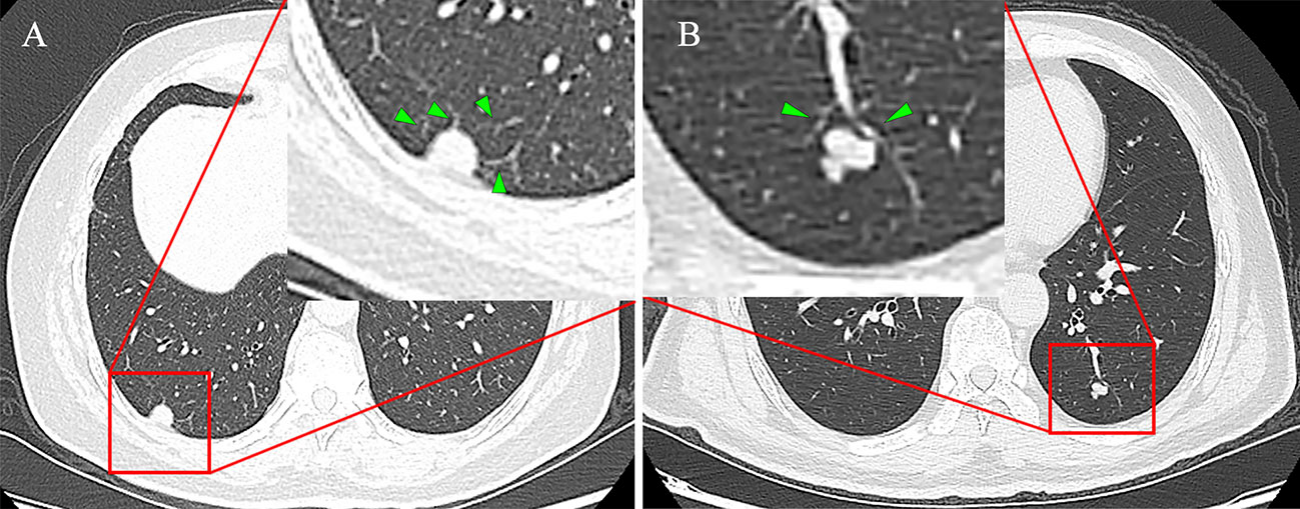
**(A)** Female, 68 years old, the SCSN had four abnormal angiogenesis into the interior of the lesion, defined as having vascular changes, no other malignant signs and pathologically confirmed adenocarcinoma. **(B)** Male, 50 years old, the SCSN had two normal vessels passing by without deformation and abnormal angiogenesis, defined as no vascular changes, and pathology confirmed as hamartoma.

CT features of the lung mass were independently evaluated by two experienced imaging physicians (with 6 and13years of experience in chest CT review, respectively) who were not informed of the pathology findings prior to the review. Disagreements between the two physicians were resolved *via* discussion. All CT findings were evaluated based on non-enhanced CT images. The main imaging features included spiculation (sunburst appearance), lobulation (abrupt bulging of the lesion contour), cavitation sign (gas-filled space presenting as a lucency or low-attenuation area), irregular air bronchogram (bronchial branch shadow with air in the nodule) and vascular change (abnormal angiogenesis or vascular distortion) (**Figure 1**), and pleural indentation (22–24).

### Histopathological analysis

Histopathological examination of surgical specimens was performed by two pathologists (with more than 10 years of experience and 15 years of experience, respectively) for pathological diagnosis. The chest CT report and clinical information were not communicated before reading the films. The excised lesions were classified according to the 2021 World Health Organization (WHO) classification (25).

### Nodule segmentation and feature extraction

RadCloud platform (version 2.1.2, https://mics.huiyihuiying.com/, Huiying Medical Technology Co., Ltd, Beijing, China) was used to manage image data, clinical data, and radiomic feature extraction.

CT images were acquired and uploaded to the database according to a standardized scanning protocol. The regions of interest (ROIs) were manually outlined on the CT images by a junior imaging physician (Dr. Song), and then all contours were examined by a senior imaging physician (Dr. zeng). If the discrepancy was ≥ 5%, the senior imaging physician determined the boundary.

Based on the ROIs outlined on CT images, 1409 features were automatically extracted using the RadCloud platform. The features were then divided into four categories (1): first-order statistics, which mainly describe the variation of voxel intensity in distributed CT images; (2) shape- and dimension-based features; (3) texture features, including grayscale dependence matrix, grayscale tour matrix, neighborhood grayscale difference matrix, grayscale size region matrix, and texture features that can quantify differences in regional heterogeneity; and (4) higher-order statistical features, which are obtained by filtering transformations on the original image. The filters used in the present study included exponential, square, square root, logarithmic, gradient, Ibp-2D, and wavelet. All imaging radiomics features were defined in accordance with those described in the Imaging Biomarker Standardization Initiative (26).

The analysis of the interclass and intraclass correlation coefficients (ICCs) was used to test the consistency of radiomics features of inter- and intra-observers. CT images of 60 patients were randomly selected for the test. To assess the reliability of inter-observers, a imaging physician (Dr. Song) segmented the ROIs, and after 1 week, the same radiologist segmented the ROIs for the second time. To assess the reliability of intra-observers, ROI segmentation was performed independently by another imaging physician (Dr. Zeng) and compared with the first segmentation results of the first radiologist (Dr. Song). An ICC value greater than 0.75 indicates good consistency and is included in the follow-up study.

Student’s t-test and the chi-square test were used to compare age, gender, tumor markers, smoking history, diabetes mellitus history, family history of malignancy, and CT characteristics between the two groups. The clinical factors (including gender and age) and subjective CT characteristics were analyzed by multifactorial regression to select meaningful indicators for the prediction model.

A large number of image features was obtained using imaging radiomics. However, not all of these extracted features may be useful for a specific task. Therefore, dimensionality reduction and selecting task-specific features for optimal performance are necessary steps. The least absolute shrinkage and selection operator (LASSO) was used to reduce the redundant features. For the LASSO model, the L1 regularizer was used as the cost function with an error value of 5 for cross-validation and a maximum number of 1000 iterations. The LASSO algorithm was used to reduce the dimensionality and select the best radiomic features to calculate the radiomic score (Ra-score) of each lesion. The Ra-score of benign and malignant SCSNs in the training and validation cohort were analyzed by Student’s t-test to clarify whether there were differences in Ra-scores between different pathological types.

Three prediction models for subjective features, Ra-score, and subjective features combined with Ra-score were built separately in the training set using logistic regression analysis. The prediction performance of the three models was evaluated using receiver operating characteristic (ROC) curves. The optimal model was then selected and validated in the validation set. Finally, the individualized prediction nomogram based on multivariate logistic model was constructed.

### Statistical analysis

Statistical analyses were performed using SPSS software 22.0(IBM, Chicago, IL, USA) and Empower software. A p value of <0.05 was considered to indicate a statistically significant difference.

## Results

The clinical data of the patients are presented in **Table 1**. Of the benign SCSNs in the training cohort 82.93% were inflammatory (including chronic inflammation and granulomatous inflammation),13.41% were benign tumors (including hamartoma, pulmonary sclerosing pneumocytoma, adenofibrolipoma and bronchial adenoma), and 2.44% were intrapulmonary lymph nodes and 1.22% were fibrotic nodules; while in the validation group, the percentages were 64.52%, 25.81%, 3.23%, and 6.45%, respectively. In the validation cohort, 93.85% of the malignant SCSNs were adenocarcinomas, 4.62% were squamous cell carcinoma, and 1.54% were metastatic tumor; while in the malignant group, all were adenocarcinomas.

Multivariate logistic regression analysis showed that vascular changes in subjective features were an independent factor in identifying benign and malignant SCSNs (**Table 2**). Based on this, a prediction model for benign and malignant pulmonary nodules (subjective model, Model 1) had an AUC of 0.672 for the ROC curve and a mediocre predictive efficacy.

**Table 2:** Multivariate analyses.

	Training cohort		Validation cohort	
	OR(95%CI)	P value	OR(95%CI)	P value
Spiculation	2.361(0.834-6.683)	0.105	3.393(0.770-14.956)	0.107
Vascular change	12.361(3.556-42.964)	<0.001	22.968(4.195-125.761)	<0.001

**Figure 2:**
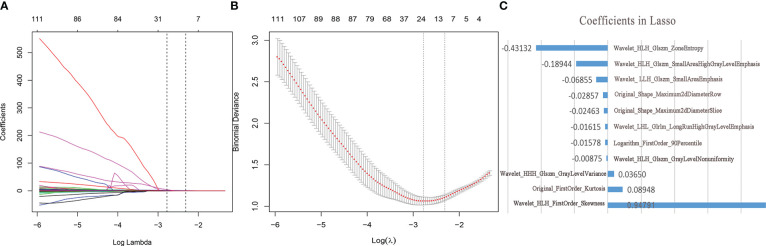
Feature selection and dimension reduction. **(A)** Ten-fold cross-validation of LASSO analysis was used to acquire the most valuable features when the minimum lambda was 0.0621. **(B)** LASSO regression coefficients **(C)** The radiological features selected by LASSO regression and its coefficients.


The imaging histology data were screened using the LASSO regression to reduce the dimensionality (**Figure 2**). A total of 11 potential predictors were selected and their Ra-scores were calculated using the following formula:

$$\begin{array}{l} \text{Ra}-\text{score}=0.08948*\text{Original}\_\text{FirstOrder}\_\text{Kurtosis}-\\ 0.02463*\text{Original}\_\text{Shape}\_\text{Maximum}2\text{dDiameterSlice}-\\ 0.02857*\text{Original}\_\text{Shape}\_\text{Maximum}2\text{dDiameterRow}-\\ 0.\;01578*\text{Logarithm}\_\text{FirstOrder}\_90\text{Percentile}-\\ 0.01615*\text{Wavelet}\_\text{LHL}\_\text{Glrlm}\_\text{LongRunHighGrayLevelEmphasis}-\\ 0.06855*\text{Wavelet}\_\text{ LLH}\_\text{Glszm}\_\text{SmallAreaEmphasis}+\\ 0.94791*\text{Wavelet}\_\text{HLH}\_\text{FirstOrder}\_\text{Skewness}-\\ 0.00875*\text{Wavelet}\_\text{HLH}\_\text{Glszm}\_\text{GrayLevelNonuniformity}-\\ 0.18944*\text{Wavelet}\_\text{HLH}\_\text{Glszm}\_\text{SmallAreaHighGrayLevelEmphasis}-\\ 0.43132*\text{wavelet}\_\text{HLH}\_\text{Glszm}\_\text{ZoneEntropy}+\\ 0.0365*\text{Wavelet}\_\text{HHH}\_\text{Glszm}\_\text{GrayLevelVariance} \end{array}$$

**Figure 3:**
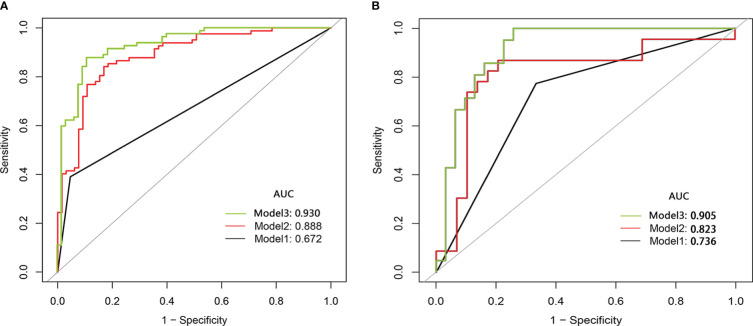
The comparison of the ROC curve of subjective model (Model 1,black line), radiomics score model(Model 2,red line)and comprehensive model(Model 3,green line) in the training cohort **(A)** and the validation cohort **(B)**. ROC, receiver operating characteristic; AUC, area under the curve.

The Student’s t-test showed no significant difference in the Ra-score of benign SCSNs between the training and validation cohort (p=0.553),as well as in the malignant SCSNs(p=0.095). Model 2(radiomic score model) was a prediction model constructed using logistic regression analysis of Ra-score. It had an ROC curve AUC of 0.888 (0.835–0.942, p<0.001) and its predictive efficacy was significantly improved. In contrast, Model 3, which was a comprehensive prediction model generated by combining the Ra-score and subjective characteristics, had an AUC of 0.930 (0.888–0.972, p<0.001) for the ROC curve. Its predictive efficacy was enhanced in comparison to Model 2, and this difference was statistically significant (**Figure 3**).

### Model validation

**Figure 4:**
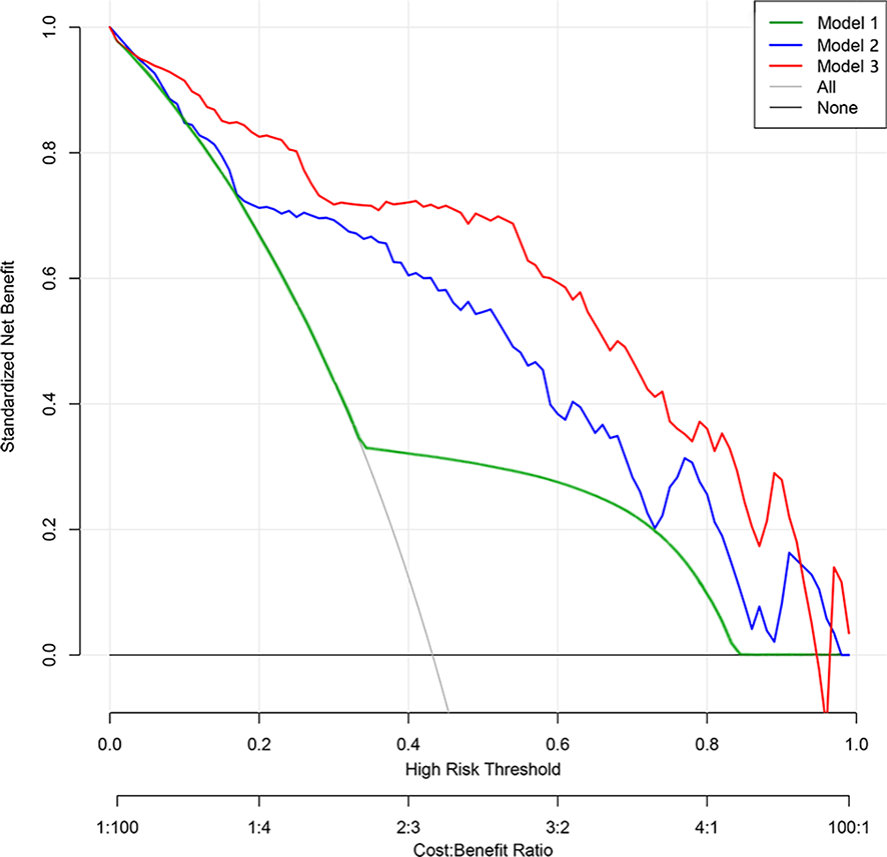
Decision curve analysis of prediction models. The y-axis represents the net benefit. The gray line represents the assumption that all patients had lung malignant nodules. The black line represents the hypothesis that all patients had benign nodules. The green line represents Model 1 (subjective model). The blue line represents Model 2 (radiomic nomogram model). The red line represents Model 3 (comprehensive nomogram). The x-axis represents the threshold probability. The threshold probability is where the expected benefit of treatment is equal to the expected benefit of no treatment. The decision curve shows that model 2 adds more net benefit than model 1, while model 3 performs better than model 2 in the range of 0.05 to 0.92.

**Figure 5:**
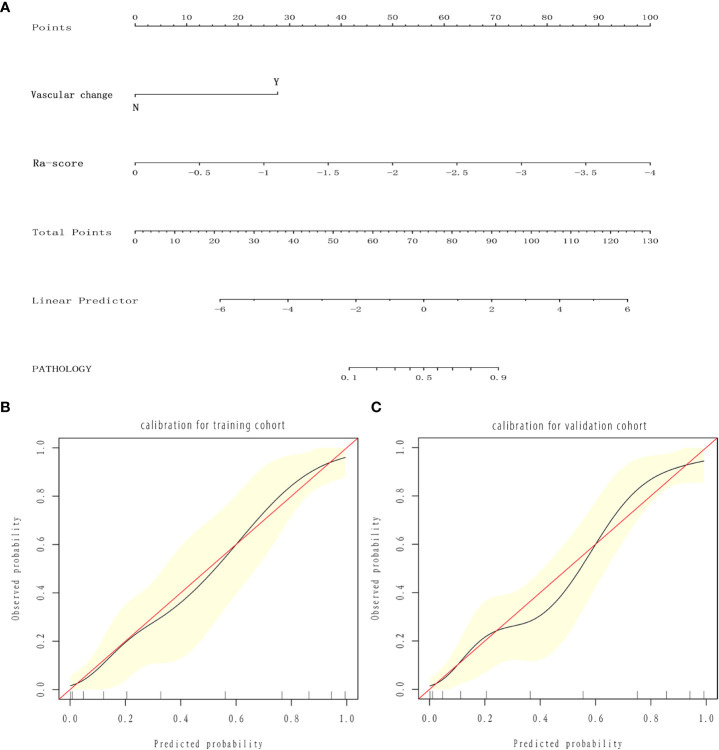
Nomogram and calibration curves. **(A)** Nomogram for predicting benign and malignant SCSNs. **(B, C)** Calibration curves of the radiomics nomogram in the training **(B)** and validation **(C)** cohort. The calibration curve describes the calibration of the nomogram based on the agreement between the prediction of the benign and malignant pulmonary nodules and the observed actual benign and malignant results. The red line represents the perfect prediction, and the red line represents the prediction performance of the radiomics nomogram. The yellow area is the 95% confidence interval (CI) of the calibration curve.

The three models were applied to a validation cohort consisting of 52 cases. The results showed that the AUC of the Model 3 ROC curve was 0.905, which was better than that of Model 1 (AUC = 0.736) and Model 2 (AUC = 0.823). Thus, the integrated prediction model based on the Ra-scores and subjective features was well stabilized and had a high predictive efficacy (**Table 3**). The DCA(decision curve analysis) showed that the overall net benefit of the integrated model column line plot was greater compared to the radiomics column line plot (**Figure 4**). A nomogram model was constructed that incorporated the radiomics score and clinical features (**Figure 5A**). The calibration curve demonstrated favorable calibration with the training and validation cohorts (**Figures 5B, C**).

**Table 3:** Predictive efficacy of models in the training and validation cohorts.

MODEL	Training cohort				Validation cohort			
	AUC	Sen	Spe	Acc	AUC	Sen	Spe	Acc
Model 1	0.672(0.613-0.731)	0.954	0.390	0.640	0.736	0.904	0.742	0.750
Model 2	0.888(0.835-0.942)	0.831	0.842	0.836	0.823	0.714	0.774	0.808
Model 3	0.930(0.888-0.972)	0.892	0.878	0.885	0.905(0.822-0.988)	0.762	0.936	0.846

AUC, area under curve; Sen, sensitivity; Spc, specificity; Acc, accuracy.

## Discussion

Pulmonary nodules are becoming more and more common in clinical experience. Even though current studies have been investigating the pGGNs and mGGNs more thoroughly, there is still not enough research on solid nodules. Moreover, all of the malignant solid nodules diagnosed in the present study were invasive carcinomas. It is not uncommon to encounter solid and micropapillary types of lung cancer with a high risk of recurrence and metastasis, which shows that malignant solid nodules can have a great impact on health if they go undiagnosed. The present study constructed a comprehensive subjective characteristics and Ra-score nomogram using logistic regression analysis to identify the benign and malignant pulmonary SCSNs. The model had a high predictive accuracy and provided a new idea for the diagnosis of SCSNs.

Multifactorial analysis showed that only vascular changes were helpful for disease diagnosis. This is likely because even though patients’ clinical information can sometimes be helpful for disease diagnosis, such as family history of lung cancer and tumor indicators, its specificity and sensitivity are not sufficient. Although conventional radiographs can suggest the benignity and malignancy of lung nodules using nodal features, such as pleural traction sign, burr sign, and lobar sign, when the nodules are small, their malignant imaging features are often not obvious and difficult to identify with the naked eye. This introduces a certain level of subjective bias, which is consistent with a previous study by Gao et al. (27). In contrast, several studies have shown that vascular changes, such as abnormal angiogenesis or vascular distortion, are commonly seen in malignant pulmonary nodules (23, 28). The area under the ROC curve of the clinical features prediction model based on this was 0.672 and had an average predictive efficacy.

In fact, CT images contain a lot of important information about lung nodules. Previously, some studies have found imaging histology to be effective in differentiating lung cancer from tuberculosis and analyzing tumor pathology types and gene expression (17–21). However, the radiological features selected in these studies were relatively few, which led to a low validity and stability of their results. In the present study, 1409 radiological features were extracted from images, including shape, size, boundary, density, and texture features. The LASSO algorithm was used to perform radiomic feature analysis in the training set, identifying 11 radiomic features with non-zero weighting coefficients. These were utilized to generate the final radiomic feature model to improve model stability. As a result, the AUC of the imaging histology prediction model was 0.888, which improved the prediction efficacy compared to the clinical feature prediction model. This difference was statistically significant. While combining imaging histology and subjective features to build the prediction model, the AUC of the ROC curve was 0.930 (0.888–0.972, p = 0.001), with a further increase in prediction efficacy that was statistically significantly different. We analyzed that the reason might be that the vascular changes were located outside of the pulmonary nodules and the abnormal vessels could not be outlined together with the nodules. Therefore, the histological imaging information did not include the vascular changes and the diagnostic efficacy was further improved by including them in the prediction model later on. Some studies have also included the peri-tumoral region, which is a 5-mm area around the nodule, in the imaging radiomics analysis after the nodule is outlined. Although this includes information about peri-nodal vessels, it cannot distinguish normal from abnormal vessels, and its application is limited when the nodule is surrounded by normal tissues, such as pleura and bronchi (29).

Methods and predictive models for discriminating the nature of lung nodules during early screening of lung cancer are increasingly becoming an important topic in the current clinical field. In previous studies, imaging radiomics and line drawings have been used to predict lymph node metastasis and prognosis in lung, colorectal, bladder, kidney, and gastric cancers, invasiveness of lung nodes, and epidermal growth factor receptor mutations (17–21, 30–33). However, there are no relevant studies on predicting the malignancy of lung SCSNs, and most of the above studies utilized pure imaging and radiomics models. The present study therefore is able to fill this gap. It also established several models based on subjective features and imaging radiomics and compared each model in order to identify the optimal model with predictive efficacy that was significantly better than that in similar studies. The t-test showed no significant difference of the Ra-scores between the different groups, indicating that although the type and proportion of pathology in the training and validation groups were not identical, it did not affect the stability of the results.

Due to the differences in clinician expertise and the lack of model understanding, it is difficult to extend the research results for lung nodule property discrimination to clinical application. Therefore, considering the prognostic significance of lung nodule characterization in early screening for lung cancer, the present study presents a nomogram of lung nodule characterization to assist physicians in diagnosing the malignancy of lung nodules, making it easy to use in clinical practice and helping to guide surgical treatment and clinical decision making.

There are some limitations in our study. First, the segmentation of CT images was manually outlined by clinicians, and although the data were reproducible by two physicians who outlined the review ROIs, subjective bias may remain. Therefore, this limits large-scale application, and the introduction of artificial intelligence to outline the ROIs should be considered at a later stage to further reduce the selection bias and facilitate data application. Second, the study data were collected from two centers in Shandong Province Qianfo Mountain Hospital and Shandong First Medical University Affiliated Provincial Hospital. Thus, these data are not as convincing as data from multicenter cohorts and different populations. Multicenter studies should be performed in the future. Third, Radiomics is inherently data-driven and lacks underlying biological principles by screening a large number of image features for reproducibility and potential information. Most published radiomics studies show no validation of the features used, except for the use of independent test sets. Yip et al. found an association between imaging features and radiomics features, but the association was not strong (34). Skogen et al. demonstrated in glioma that tumor grading, which is known to correlate with heterogeneity, correlated with the standard deviation of intensity distribution in CT images (35). In contrast, Liu et al. reported no correlation between tumor grading and heterogeneity, while showing a strong association with textural features (36). More studies may be needed to verify the biological meaning of the radiomics features. This disconnect between predictive models and biological significance will inevitably limit its wide clinical application. Combining clinical and preclinical experiments may also play an important role in the biological validation of imaging histology. Animal studies enable the experimental interventions necessary to establish causal relationships between biology and imaging histology and to provide precise, spatially aligned histological analyses for in-depth validation. We hope to do deeper research in future studies. Fourth, our study was a retrospective analysis of patients with pulmonary SCSN who underwent surgery at two centers, but in reality, a proportion of patients with pulmonary SCSN did not undergo surgery but were observed at long-term follow-up, which led to selective bias and a situation that was similar in some other studies (37, 38). In future studies, we will increase the sample size to include cases that did not undergo surgery with further follow-up observations to further validate and optimize this prediction model.

In conclusion, the imaging of histological features of plain CT images may help to identify benign and malignant SCSNs. A nomogram based on the imaging of histological features and abnormal vessels can be an effective tool to diagnose benign and malignant sub-centimeter solid pulmonary nodules and thus guide clinical decision making.

## Data availability statement

The original contributions presented in the study are included in the article/Supplementary Material. Further inquiries can be directed to the corresponding author.

## Ethics statement

The studies involving human participants were reviewed and approved by Ethics Committee of the First Affiliated Hospital of Shandong First Medical University, Ethics Committee of the Provincial Hospital of Shandong First Medical University. Written informed consent for participation was not required for this study in accordance with the national legislation and the institutional requirements.

## Author contributions

QSZ and GW contributed to the conception of the study; QG and CC performed the experiment; QG contributed significantly to analysis and manuscript preparation; CC performed the data analyses and wrote the manuscript; ZD and YW assisted in the development of the radiomics model and data analysis; QZ, DS, and GS helped perform the analysis with constructive discussions. All authors contributed to the article and approved the submitted version.
